# Attitudes Toward Aging, Physical Activity, and Functional Limitations Ten Years Later in Middle and Older Adults

**DOI:** 10.1093/geroni/igaa057.3356

**Published:** 2020-12-16

**Authors:** Yen Chen, Carole Holahan, Charles Holahan

**Affiliations:** The University of Texas at Austin, Austin, Texas, United States

## Abstract

Positive attitudes toward aging have been associated with better functional health (Bryant et al., 2012; Levy, Slade, & Kasl, 2002; Sargent-Cox, Anstey, & Luszcz, 2012). The current study examined the mediational role of regular leisure-time physical activity (LTPA) in this association among middle-aged and older adults. Participants were 2,209 adults ranging in age from 40 to 75 at baseline (M = 56.19; 51.2% women) from the second and the third waves of the Survey of Midlife Development in the United States (MIDUS). The mediation model was tested in Mplus 7.4. The model tested the direct association and indirect association through LTPA of attitudes toward aging in MIDUS 2 with functional limitations in MIDUS 3 ten years later. Attitudes toward aging were measured as a latent variable with two indicators (subjective age and future health expectancies). Age, sex, race/ethnicity, education, marital status, and number of chronic conditions were included as covariates. Individuals with more positive attitudes toward aging reported less functional limitations ten years later (β = -.43, p < .001). Further, more positive attitudes toward aging related to higher levels of LTPA (β = .20, p < .001), which in turn predicted less functional limitations ten years later (β = -.10, p < .01). The indirect effect of attitudes toward aging on functional limitations through LTPA was statistically significant (indirect effect = -.02, p < .01). Positive attitudes toward aging operating through higher levels of LTPA may play an important role in functional health among middle-aged and older individuals.

